# New products

**DOI:** 10.1155/S146392468600010X

**Published:** 1986


Journal of Automatic Chemistry/*Journal of Clinical Laboratory Automation*, Volume 8, Number 1 (January–March 1986), pages 36–44

### New products

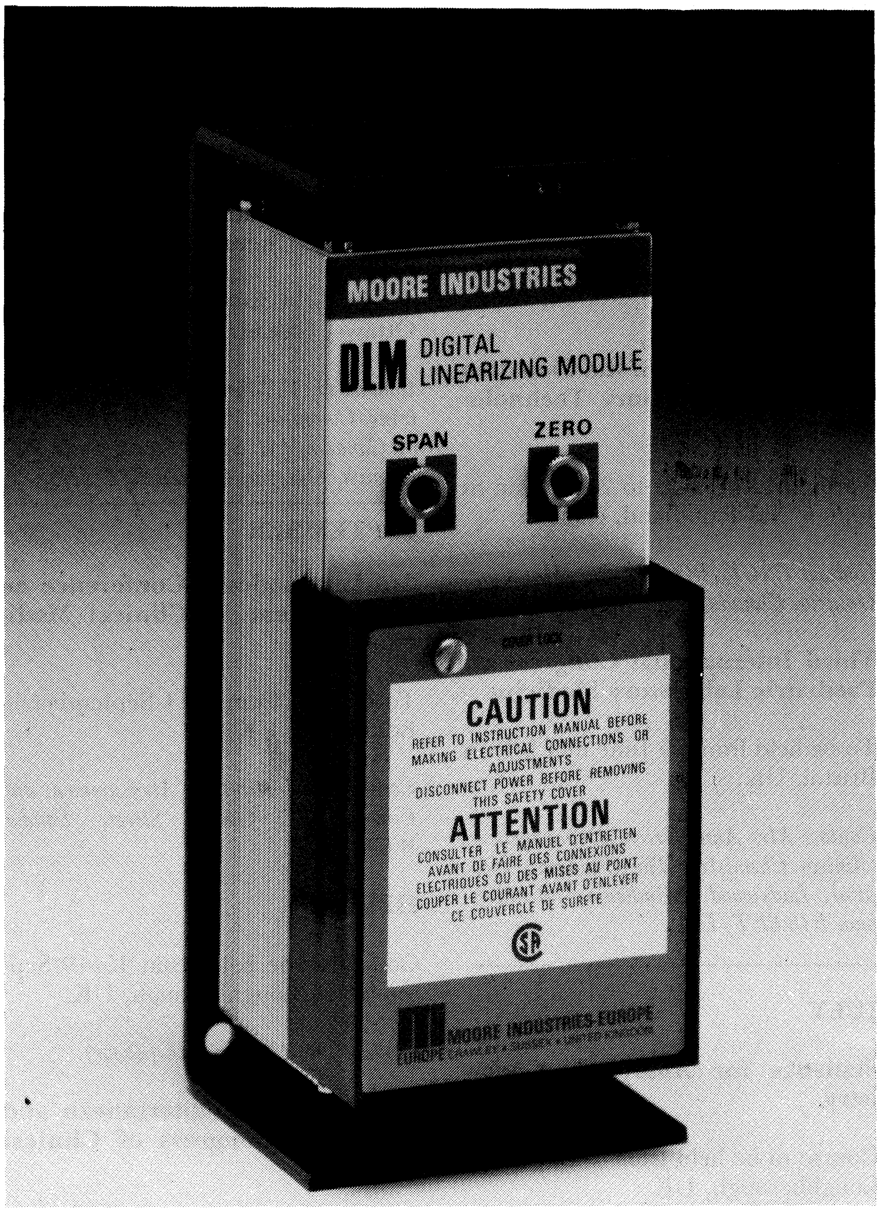

*New compact digital linearizer modules with EPROM memories programmed for particular applications are now available from Moore Industries. The modules (DLMs) are used to convert non-linear voltage or current inputs into linear voltage or current outputs.*

*The characteristics of each DLM supplied are held in the Moore Industries computer so that customers' orders may be reproduced as required and new programs can be burnt in to suit different applications.*

*The DLM will handle any non-ambiguous input curve without restrictions on the number of slope or signal reversals or the steepness of the slope. Accuracy is claimed as ±0·1% span and repeatability ±0·01% span; input resolution is stated as being 0·055%. The modules accept inputs of 1–5, 4–20, 0–20 mA, or 0–1, 0–5, 1–5 V. Linear outputs are produced in the same ranges. The unit also provides input and output isolation as a standard feature. An optional range of millivolt inputs is available and RF interference protection can be provided. More information from Moore Industries Europe, Inc., Baird Close, Maxwell Way, Crawley, West Sussex RH10 2SY, UK.*

**Circle No. 1 on Reader Enquiry Card**

### Monarch

A brochure is now available from Instrumentation Laboratory about the new Monarch chemistry system. The system provides cost-effective, fully automated analysis of up to 98% of the tests currently performed in the clinical laboratory, including electrolytes, routine chemistries, special proteins, and therapeutic drugs.

The system combines an on-board capability of 23 tests (using ISE) with quick, easy changeover to additional IL-developed tests and applications for EIAs, FIAs and special chemistries. It provides a programmable capacity of up to 100 tests, and a throughput of up to 600 tests/h, with minimal operator involvement.

The Monarch system uses multiple methods of detection, including absorbance, fluorescence, light scatter, and potentiometry, and offers a choice of test modes: random access patient-prioritized, time-optimized, and stat sampling.

*For a free copy of this colour brochure, write to Instrumentation Laboratory, Inquiry Department, 711 Forbes Avenue, Pittsburgh, Pennsylvania 15219, USA. Tel.: 617 861 0710.*

**Circle No. 2 on Reader Enquiry Card**

### Electrophoresis

A new capability in electrophoresis preparation systems was introduced by Ultra-Violet Products Ltd at the September Laboratory-85 exhibition in London. UVP is streamlining its service to users in the biotechnology field by offering complete DNA/RNA electrophoresis preparation packages. These systems, which are tailored to suit individual users' needs, can include a variety of transilluminators, camera systems, films, safety equipment, gel producing apparatus, gel dryers and power supplies.

*Full details from Ultra-Violet Products Ltd, Science Park, Milton Road, Cambridge CB4 4BN, UK. Tel.: 0223 355722.*

**Circle No. 3 on Reader Enquiry Card**

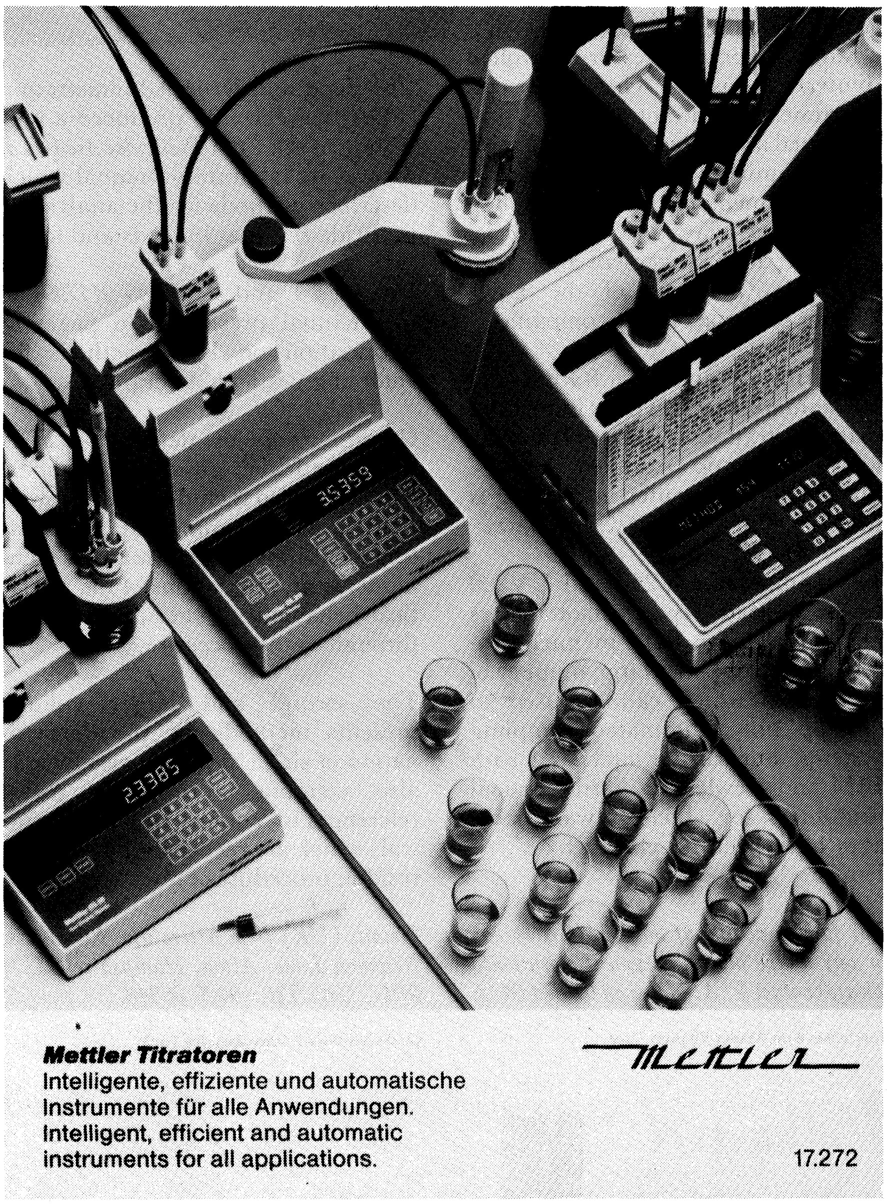

*A brochure published by Mettler introduces the company's three compact titrators and describes their possible applications. Called 'Mettler Family of Titrators' the booklet also contains information about the many connection and expansion possibilities of Mettler titrators. It shows the connection of balances with automatic processing weighing data, printers and printer-plotters, as well as the use of Mettler titrators in combination with sample transports, robots and Kjeldahl distillation instruments. Finally, the brochure describes such titration accessories as electrodes, titration vessels and burettes. Particularly noteworthy in this respect are the phototrodes for calorimetric end-point indication. For more information contact Ruedi Schulthess, Mettler Instrumente AG, CH-8606 Greifensee, Switzerland. Tel.: 941 22 41.*

**Circle No. 4 on Reader Enquiry Card**

### Thermometer

Automatic Systems Laboratories has introduced a new precision thermometer. The F25 gives a stable measurement for one or two PT100 resistance probes, displaying both absolute and differential temperatures. Its absolute accuracy is ±0·025 °C with user-selectable resolutions of 0·01 °C and 0·001 °C over the full temperature range of −100 °C to +450 °C. This range can be extended by using alternative probes.

The F25 is very easy to use, and its high performance results from further developments in ASL's established AC bridge techniques which have been proven in standards laboratories throughout the world. This ensures an instrument requiring no recalibration and which is free of the drifts and thermal EMF errors associated with d.c. carrier systems.

A clear display indicates in either °C or °K and can be set to zero at the touch of a button, for either probe input or differential read-out.

A range of robust and economically priced calibrated PT100 probes is available for use with the new thermometer. Linearization data is held internally within the instrument's microprocessor. The use of calibrated PT100 probes means that the measurements are traceable to national standards.

The company says that the F25 offers exceptional performance at an economic cost, particularly as an RS232 interface for both data acquisition and instrument control is included as standard. An IEEE interface is available as an optional extra.

It is understood that, for multi-channel applications, ASL will soon be bringing out a new scanner designed specifically for the F25.

*More information from ASL, Saxon Street, Linford Wood, Milton Keynes, Buckinghamshire MK14 6LD, UK.*

**Circle No. 5 on Reader Enquiry Card**

### V. A. Howe

V. A. Howe and Company, as the UK's largest independent suppliers of laboratory equipment, are able to offer an extensive range of instruments which can enhance the degree of automation achieved in a laboratory.

Of particular note is the AMICA system developed by Hamilton of Switzerland in close co-operation with Ciba-Geigy. This system was designed to solve problems of chemical and pharmaceutical industries which are in need of automation for chemical analyses. AMICA combines the features of several analysers and performs its tasks consistently, quickly and economically. The system's capabilities include sample preparation, automatic sampling, reactant pick-up from any liquid container, continuous sampling, mixing of liquids in variable ratios and analysis in flow-through cell by various techniques. These can include spectrophotometry, potentiometry and amperometry. User-friendly dialogue is exhibited by a microcomputer, as well as logging of data and results by a printer/plotter.

The system is particularly useful for quality control analysis in the pharmaceutical industry. Sample data and results, together with statistical analysis, are logged as a permanent record by the printer/plotter.

The purity of many chemicals and pharmaceuticals is often determined by non-aqueous titrations of weak organic acids, frequently using tetrabutylammonium hydroxide as titrant. AMICA is ideal for this application for several reasons: it is a closed liquid system and, as such, no special precautions are required to avoid either the absorption of $CO_2$ from, or the release of solvents into, the atmosphere. AMICA also performs the titrations very accurately and rapidly, typical analysis time being under 2 min per sample, with both accuracy and reproducibility at around 0·2%.

In the food industry, for instance, determination of nitrite, by photometric analysis, is undertaken frequently when sodium nitrite has been used to preserve red meat constituents.

During the manufacture of printed circuit boards, acidic electroplating baths of copper and tin are used. The quality of the products is dependent upon the accurate control of both acid and metal concentrations of these baths and these analyses are often performed by titration.

The Hamilton AMICA system is able to determine the concentration of these constituents in either discrete sample form or by on-line analysis. After rapid analysis, data is printed out, but AMICA could also command a process computer to automatically adjust the strength of the bath.

Also available from Hamilton are a range of microprocessor-controlled diluter/dispensers. These versatile instruments can not only be programmed and operated through their control units which have battery-protected memory, but also through a computer via an RS232C serial interface: an arrangement already in operation with one of the UK's leading pharmaceutical companies.

V. A. Howe as agents for Radiometer of Denmark can also offer fully automated titration systems complete with automatic sample changes. Once again, computer interfacing is possible with an RS232C interface.

The Shimadzu UV240 and UV260 research grade spectrophotometers not only feature built in microprocessor control and a large number of accessories which can be used to develop fully automated sampling systems, but can be interfaced with IBM and Hewlett-Packard personal computers, for which software packages have been developed by V. A. Howe.

*For further details of the AMICA system or any of the other instruments mentioned please contact V. A. Howe on 01 874 0422.*

**Circle No. 6 on Reader Enquiry Card**

### Pesticides in food

The Food and Drug Administration (FDA) has recently produced a new edition of the *FDA Pesticides Analytical Manual*—a laboratory manual which describes methods for the analysis of pesticide residues in foods and feeds.

Volumes 1 and 2 (*PB85–911799*) of the manual present 2878 pages of information on FDA methods for enforcing pesticide tolerances.

Giving complete details of FDA multi-pesticide residue methods, Volume 1 also includes sample preparation, gas chromatographic operating conditions, confirmatory techniques, and tabulations of the analytical behaviour of various chemicals through each procedure.

The second and larger volume presents methods for the determination of single pesticide residues. It also contains appropriate cross-references to Volume 1 for the chemicals which are recovered by multiple residue procedures.

*Details (UK) from Microinfo, PO Box 3, Newman Lane, Alton, Hampshire OU34 2PG, UK. Tel.: 0420 86848.*

**Circle No. 7 on Reader Enquiry Card**

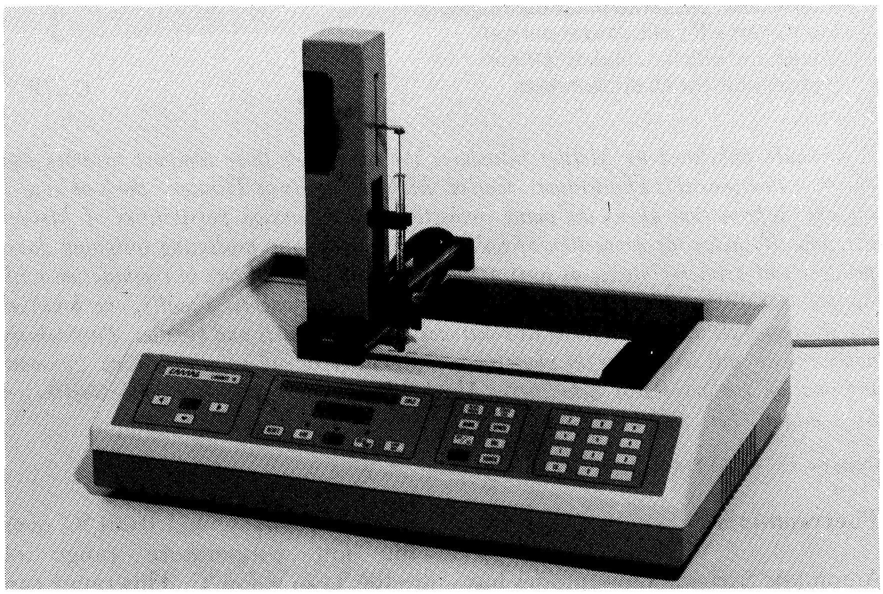

*CAMAG has introduced the LINOMAT IV, a microprocessor-controlled, programmable instrument with keyboard parameter entry. From band length, distance between samples, edge space, and plate width, the instrument computes the number of available tracks and indicates them by means of light-emitting diodes. Track location and application of several sample deliveries from the syringe proceed automatically. Details from Ch. Gfeller, CAMAG, Sonnenmattstrasse II, CH 4132 Muttenz, Switzerland. Tel.: 061 61 34 34.*

**Circle No. 8 on Reader Enquiry Card**

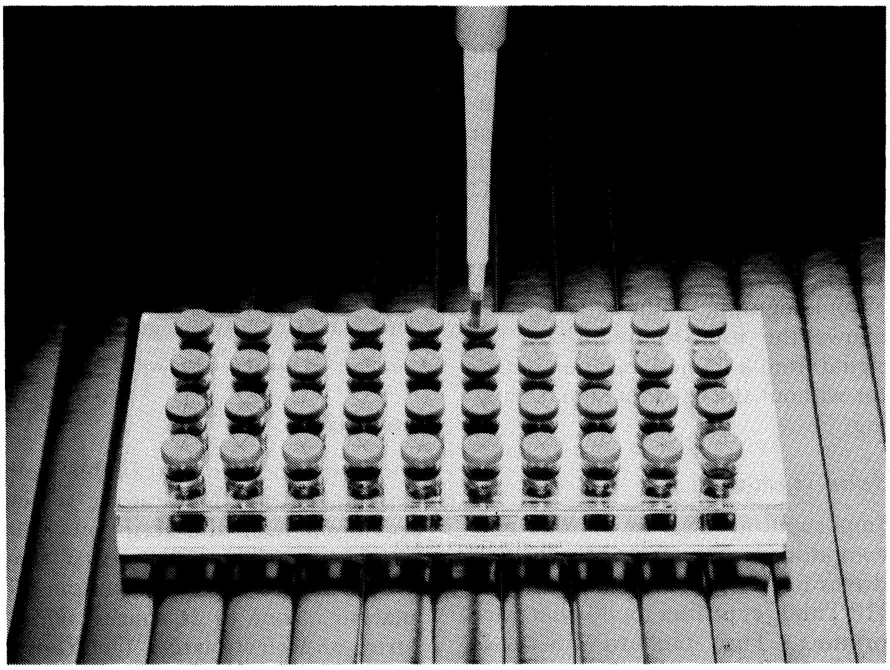

*Elkay have introduced the Ezee-Perf cap to fit all Elkay sample cups up to 2.5 ml including the SMAC type cup. The cap, which is precision-moulded from low-density polyethylene, is perforated to allow analyser probe penetration or pipetting without removing the cap. The Ezee-Perf cap is also ideal for short-term storage, as it minimizes the possibility of contamination. When dealing with paediatric samples, the Elkay cap is recommended to help minimize evaporation prior to sample aspiration. Details from Elkay Laboratory Products (UK) Ltd, Unit 2, Crockford Lane, Basingstoke, Hampshire, UK.*

**Circle No. 9 on Reader Enquiry Card**

### In-process colour monitoring

Qual-Probe is an automatic, in-process colour measurement system. Qual-Probe improves the user's ability to obtain consistent colour in a variety of products—the system enables the user to monitor and control colour and colour-related properties faster and with more precision than has been previously possible.

Qual-Probe combines a solid-state fibre optic sensor with a compact wall-mounted operator station. Both components meet scrub-down requirements, NEMA/IP standards and are not affected by heat, humidity or vibration.

The sensor is positioned above the product as it exits the process line and can be mounted either in a stationary position or on a traversing assembly. This positioning flexibility allows the user to tailor the system to conform to varying product sizes, shapes and flow patterns. The operator station is designed for simplicity of use and can support up to three sensors, for use on separate production lines, or for example, for monitoring top and bottom colour on the same line. The operator station allows permanent storage of previously determined color standards and acceptable limits (tolerances) for different products. It displays a single number to represent the colour value of the product.

*Details from HunterLab, Process Monitoring and Control Division, 11495 Sunset Hills Road, Reston, Virginia 22090, USA. Tel.: 703 471 6870.*

**Circle No. 10 on Reader Enquiry Card**

### Infra-red analyser

The IRGA 320 non-dispersive infra-red analyser makes the high sensitivity and selectivity of the well-proven IRGA 220 laboratory instrument available to industrial markets. The analyser continuously measures the concentration of one gas in a mixture and provides wide measuring ranges for many gases and vapours (CO, $C_2$, CH4, $N_2O$, hydrocarbons and refrigerant gases for example). It can be calibrated for two independent measuring ranges with a dual linear scale meter. Repeatability is ±1% of full scale deflection or better, linearity is ±2% or better, and response time is typically less than 10 s for most samples.

The IRGA 320 is packaged in Sieger TPA's modular system in sealed dust-proof units, which can be purged for electrically hazardous atmospheres. Options include a digital display with auto-zero unit, a dual level alarm, and full microprocessor control through which the sample information can be converted to customer-defined parameters via a plug-in keypad.

*More information from Sieger TPA Ltd, 34 Tresham Road, Orton Southgate, Peterborough PE2 0SE, UK. Tel.: 0733 235500.*

**Circle No. 11 on Reader Enquiry Card**

### Computing integrator

The new HP3393A computing integrator from Hewlett-Packard adds advanced software and built-in functionality to the proven precision of the HP3390 integrator series, and provides extensive data-handling capabilities for the analytical laboratory. This new system not only integrates, calculates and reports, it also re-integrates, replots, performs multi-level calibration and provides BASIC programming language.

To give HP3393A full functionality as a computing system, an alphanumeric typewriter-style keyboard, a thermal printer/plotter and RS-232-C, INET (instrument network) and sample number-input communication/control interfaces are provided as standard, built-in features.

The HP3393A computing integrator system takes advantage of proven technology and reliability improvements made during the five-year history of the HP3390 family.

Once the chromatographic signal has been transmitted to the integrator, it can be stored locally or in external disk memory. It then can be re-integrated, replotted and recalculated as many times as necessary at speeds much faster than the original run. This is extremely helpful in cases where only a small amount of sample is available or during the development of a method for an unfamiliar sample.

Multi-level calibration enables users to match the calibration to their chemistry, ensuring accurate results. In addition to traditional single-point calibration, HP3393A offers linear regression, non-linear (quadratic), or point-to-point calibration curves.

Simple question-and-answer preparation dialogues make setting up calibration and sequence tables an easy process, especially for the infrequent user. Unlike most other integrators, there is no need to memorize commands.

*Programming*

The HP3393A Basic programming language is an interactive compiler that executes at speeds about 10 times faster than traditional interpreted Basic.

A set of 231 commands, statements and functions allows for flexible control over all aspects of the chromatographic system, including post-run calculations and custom reports. Interactive control over chromatography and automatic-sampler parameters also is possible.

The alphanumeric keyboard reduces the need for manual record-keeping, minimizing interpretation errors and simplifying peak identification. Calibration and data files can be assigned names, not just numbers. The built-in keyboard also can be used to create and edit Basic programs.

*INET configuration*

As part of Hewlett-Packard's instrument network (INET), the computing integrator can serve as a controller for an HP5890A gas chromatograph, HP1090A liquid chromatograph, HP7673A automatic sampler/injector and HP19405A event control module.

In such a configuration, the operating parameters of all the instruments on the network are stored on disk or in the HP3393A, which has battery back-up in case of power failure. All set points and parameters for each instrument on the communications loop can be activated directly from the integrator's keyboard. Most parameters can be changed on a run-to-run basis through a BASIC program.

When used with the HP5890A gas chromatograph, the integrator allows the user to quantitate the signal from a flame-ionisation detector without the need for signal ranging.

*System expansion*

Important accessories to the new computing integrator include disk drives for increased storage and the HP ThinkJet printer for 80-character printing. The standard RS-232-C interface allows communication with a work-station, personal computer or laboratory data system.

*Readers' enquiries to Enquiry Section, Hewlett-Packard Ltd., Eskdale Road, Winnersh, Wokingham, Berkshire, UK.*

**Circle No. 12 on Reader Enquiry Card**

### Spectrometry information

Hilger Analytical has published the first four in a series of technical information sheets: *'Polyvac at Work'*.

The Polyvac series of spectrometers have become well known for their performance and quality throughout the world, analysing rapidly and accurately elemental composition of raw materials or manufactured products.

The first information sheet is called 'Carbon gradient in case-hardened steel' and describes the QC testing of components to ensure that they have been case-hardened correctly.

The second, 'Nickel-based alloys', deals with the development of super-alloys and the precise analysis that is required.

'Free-cutting steels' is the title of the third sheet, which looks at the analytical problems encountered with free-cutting steels and the solutions achieved using the Polyvac.

The fourth publication, 'Stainless steels' embraces the analytical requirement of the AOD process, (Argon Oxygen Decarbonizing) and discusses the background and development of micro-duplex steels.

*Further information from Richard W. G. Aslet, Marketing Manager, Spectrochemistry Group, Hilger Analytical Ltd., Westwood, Margate, Kent, UK. Tel.: 0843 225131.*

**Circle No. 13 on Reader Enquiry Card**

### Genesis

A colour brochure is available from Instrumentation Laboratory, which provides a detailed description of the Genesis 21 clinical chemistry analyser. The system provides automatic analysis of one to 21 routine chemistries and electrolytes. The 21-test main menu is supplemented by a second menu for an actual capability of 36 different analytes. The system performs up to 21 simultaneous tests on any sample, and is preprogrammable for up to 50 patient samples per run.

*For a free copy of the brochure, phone Instrumentation Laboratory on 617 861 0710, or write to Instrumentation Laboratory, Genesis Marketing Coordinator, 113 Hartwell Avenue, Massachusetts 02173, USA.*

**Circle No. 14 on Reader Enquiry Card**

### Calibrator kits

Using one of the new Coulter S-Cal calibrator kits, an operator can calibrate an S or S-Plus Series Coulter Counter instrument, with daily results monitored using the company's 4C Control product. S-Cal Calibrator values for WBC, RBC, MCV and Plt parameters are derived by replicate analyses of fresh whole blood samples on several correctly calibrated S or S-Plus Series instruments. These assigned values are traceable to reference methods with an accuracy within ±2%. In addition, the accuracy of each assay instrument is verified daily with fresh whole blood samples against referenced methods as detailed on the assay sheet.

*For further information contact Coulter Electronics Ltd, Northwell Drive, Luton, Bedfordshire LU3 3RH, UK.*

**Circle No. 15 on Reader Enquiry Card**

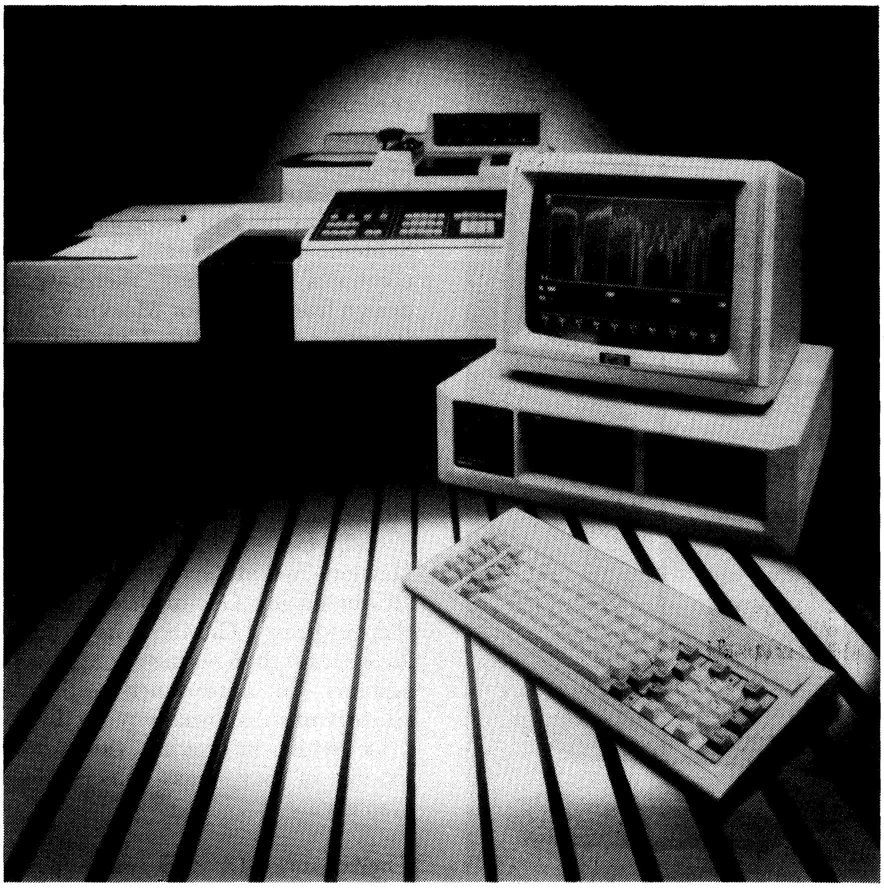

*In a move towards greater professionalism and productivity in the laboratory, Philips Analytical has launched its new data station and the first in an associated series of integrated data systems – for infra-red spectroscopy. The company has adopted as its data station the industry standard IBM PC environment which offers customers the reassurance of having support and room to grow, rather than the uncertainty of being locked into the dedicated computer products of one analytical instrumentation manufacturer. An immediate benefit is that users can take full advantage of a range of business software, including Symphony, Framework, Lotus 123 and Wordstar, in order to improve the administration and presentation of their laboratory and its work. Details from Pye Unicam, York Street, Cambridge CB1 2P4.*

**Circle No. 16 on Reader Enquiry Card**

### Blood coagulation measurement

Labsystems have launched the FP910: a new automatic system for blood coagulation measurements. Both conventional and chromogenic substrate test methodologies can be carried out with the system. The FP910 is a compact, microprocessor-controlled analyser, designed to measure and automatically evaluate end-point and kinetic tests. The instrument enables simultaneous measurement of nine samples in a cuvette block, based on the principles of vertical photometry. The software allows both conventional and chromogenic substrate tests for coagulation to be carried out quickly and reliably, including PT, APTT, AT III, Heparin, factors and inhibitors. The FP910 is compatible with reagents from a variety of maufacturers, including the 'Manchester' reagents. In PT and APTT determinations the use of the Labsystems FP900 nine-channel pipette, with an electronic starter, ensures simultaneous starting of the reaction in all channels.

The system has a high throughput (100–150 PT and APTTs/h, 200 kinetic tests/h) and is priced at around £10 000.

*More information from Labsystems (UK) Ltd, 12 Redford Way, Uxbridge, Middlesex UB8 1SZ, UK.*

**Circle No. 17 on Reader Enquiry Card**

### Variable wavelength UV detector

Beckman have announced a new UV detector—the Model 163—which provides selection of any wavelength from 195 to 350 nm in 1 nm increments. Sensitivity is a feature of the instrument.

The Model 163 is expected to be welcomed for a variety of applications, including microbore, high speed and preparative chromatography.

The entire flow cell assembly of the Model 163 can be easily removed as a unit for replacement purposes or for service. In addition to the standard analytical flow cell, a micro flow cell (1.4 μl) and a semi-preparative version (35 μl) are available.

*More information from Beckman Ltd, Progress Road, Sands Industrial Estate, High Wycombe, Buckinghamshire, UK. Tel.: 0494 41181.*

**Circle No. 18 on Reader Enquiry Card**

### Low-pressure mixing for HPLC

Low-pressure mixing is an economical way of adding binary gradient elution performance to existing isocratic HPLC systems. The new Model 1020 low pressure mixing module from Drew Scientific incorporates an electronically operated valve which proportions %B in a binary solvent mixture. It is powered and controlled by the Drew Model 1025 gradient controller. Its small overall size allows the module to be conveniently positioned between the solvent reservoirs and the single high pressure pump.

The solvent pathways are chemically inert and introduce no additional dead volumes to the chromatography system. The gradient output is within the specification of the Model 1025 gradient controller. In linear gradients, the coefficient of linearity is better than 0.999. The performance is equally good with non-linear gradients. The valve actuator frequency is adjustable to ensure that the Model 1020 is compatible with all current designs of HPLC pumps.

*More information from Drew Scientific Ltd, 12 Barley Mow Passage, Chiswick, London W4 4PH. Tel: 01 995 9382.*

**Circle No. 19 on Reader Enquiry Card**

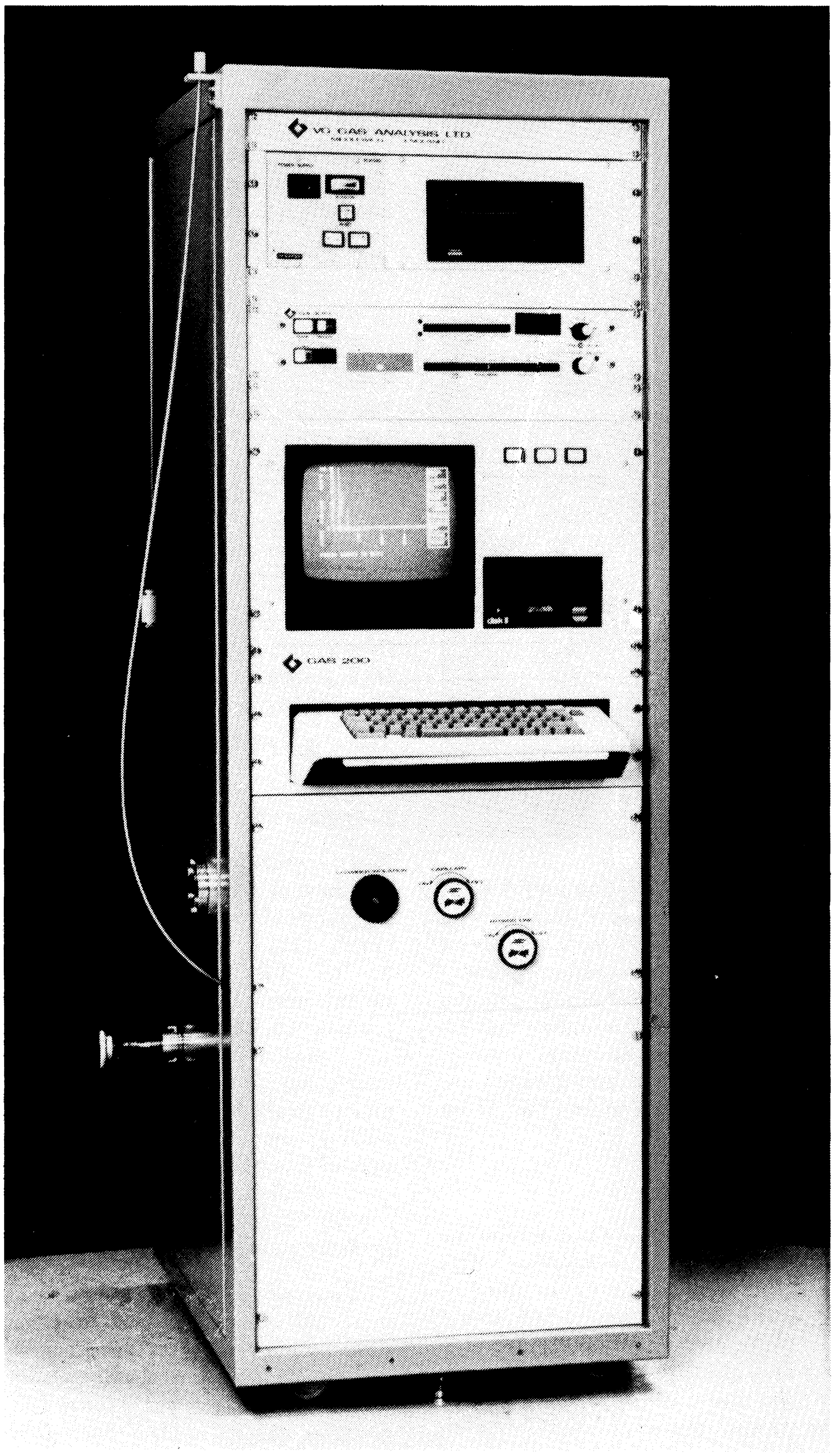

*The Systems Division of VG Gas Analysis has introduced a 1–200 amu, computer-controlled, quadrupole mass spectrometer, gas analysis system suitable for laboratory, pilot plant and on-line application. The system allows complete analysis of multi-component, multi-stream gas samples with composition output under manual or computer control. The integrated instrument is in a mobile 19 in cabinet with flexible gas inlet line, 9 in VDU, imag-writer hard copy and analogue outputs and user-programmable disk-based software, which can be formatted to cover a variety of day-to-day and special or occasional gas analyses. A choice of inlet systems and mass ranges is available. Contact Graham Atkin, VG Gas Analysis Ltd, Middlewich, Cheshire CW10 OHT, UK for further details.*

**Circle No. 20 on Reader Enquiry Card**

### Thermospray

A fully integrated thermospray liquid chromatography/ mass spectrometry system was launched by Hewlett-Packard at the 10th International Mass Spectrometry Conference, which was held in Swansea, UK in September last year. The system combines the latest thermospray design from Professor Marvin Vestal (whose group at the University of Houston first developed the thermospray ionization source as a practical LC/MS interface), with HP's technology in mass spectrometry, liquid chromatography, data handling and automation. According to Arthur Wood, UK General Manager of Hewlett-Packard's Analytical Instrumentation Group, 'the new HP thermospray LC/MS costs at least 25% less than other comparable systems, in accordance with our strategy of substantially reducing the price while improving the performance of our mass spectrometer systems'.

Thermospray LC/MS combines the separation capabilities of HPLC with the ability of the thermospray device to ionize a range of thermally labile, or non-volatile compounds, that can then be analysed by the sensitive mass spectrometric method.

The thermospray LC/MS technique has been successfully used to analyse many classes of compound, including underivatized amino-acids, urea pesticides, peptides, vitamins, glucuronides, glycols, polyglycols, organic acids, nucleotides, nucleosides and alkaloids.

Operation of the system is simple and quick. To set up, the thermospray LC/MS is tuned and calibrated with just a few keystrokes. System control and data handling are effected via an interactive analytical work-station, and several configurations are available. Users may choose the multi-user, multi-tasking, multi-instrument HP 1000 RTE-6/VMs. All provide screen-based formats for method building, data edition, calculations and report generation.

Users may tailor and expand the system using a number of optional peripherals, including a kit that converts the system from LC/MS to

GC/MS. The system is offered with or without the HP 1090 liquid chromatograph, allowing the purchaser to interface it to an exciting HPLC instrument if required.

As well as performing as a standard thermospray system, the HP thermospray LC/MS operates in several other modes. The ion source includes a rugged filament that works in either 'filament on' or 'filament off' mode. For some compounds, thermospray mass spectra can be obtained in the 'filament off' mode. Many other compounds, however, require 'filament on', either to provide more structural information through electron-assisted ionization or to increase the sensitivity.

Positive chemical ionization (CI) is standard on the HP thermospray LC/MS system for maximum specificity, while negative CI is offered as an option. Negative CI provides high sensitivity for compounds with electro-negative sites, such as fluorinated or chlorinated pesticides.

The standard HP thermospray LC/MS has a mass range of 10–1000 amu. A high mass option is available – doubling the range to 2000 amu.

The system can also be operated in the RF-only mode as a true total ion monitor (TTI), providing a sensitive universal detector for HPLC. In the TTI mode, the system detects compounds eluting from the HPLC that are above the scanning range of the mass spectrometer.

*Enquiries to Tina Mears, Analytical Instrumentation, Hewlett-Packard Ltd, Miller House, The Ring, Bracknell, Berkshire RG12 1XN, UK. Tel.: 0344 424898.*

**Circle No. 21 on Reader Enquiry Card**

### Micro-grinding

Homogenous samples of even the most difficult materials can be prepared with the Braun Micro-Dismembrator, which is available in the UK from FT Scientific Instruments of Bredon. The unit, which is based on the ball mill principle, is designed to function from well beyond the simpler duties required in inorganic sampling to the most exacting medical requirements. Sampling of hair, skin tissue, liver, kidneys – even cartilage and dental enamel – can all be achieved. It is also ideal for contamination-free preparations in trace-element analysis, dry or wet grinding, embrittling technique (grinding of frozen sample in the vial), and any other application where small samples have to be mixed, dispersed, emulsified, dissolved or extracted.

The ball mill principle allows a high kinetic energy to be generated. A vial containing the sample and grinding balls is oscillated at mains frequency; the balls are accelerated to resonate with this frequency and the high kinetic energy is used to disintegrate and mix homogenously the ample material.

Vials are available in stainless-steel or Teflon. Grinding balls, in 1 mm, 3 mm, 5 mm and 10 mm sizes are supplied in Teflon-coated steel, stainless-steel, chromium steel, tungsten carbide and agate quartz.

*Full details from FT Scientific Instruments Ltd, Station Drive, Bredon, Tewkesbury, Gloucestershire GL20 7HH, UK. Tel.: 0684 72425.*

**Circle No. 22 on Reader Enquiry Card**

### Gas analysis

GOW-MAC is offering Continuous Gas Analyzers, Models: 50–350, 50–450, 50–250, 50–150, custom designed for high sensitivity. Using thermal conductivity for accuracy, all models feature a self-contained gas-flow system, purgable cases, and sample and reference rotameters with pressure regulators. Zero stability for most applications is 1% of full-scale in a 24 h period, while the accuracy is also 1% of full scale. Applications include continuous monitoring of gas streams in static systems, atmosphere control and quantitative analysis of binary process streams of industrial gases.

Five factors are taken into account for custom applications: detector design and construction, cell-current regulation, temperature regulation, gas-flow system and read-out equipment. Single and two pass analysers may be specified. Options include: alarm contacts, eight-filament cells, 10X signal amp, digital meter, flowmeters, etc. GOW-MAC's continuous gas analysers may be either rack mounted or installed on a bench.

*By filling out an Applications Data Sheet, a customer may request specific applications. GOW-MAC will determine the suitability of the instrument to the applications. For detailed informatin, specifications and prices contact GOW-MAC Instrument Company, PO Box 32, Bound Brook, New Jersey 08805, USA.*

**Circle No. 23 on Reader Enquiry Card**

### GC

The Model 3500 GC from Varian is described by the company as providing the fastest results over a wider range of applications and the best retention time reproducibility of any other instrument on the market.

The 3500, designed specifically for use with the more inert capillary fused silica columns, was developed in response to the growing popularity of capillary gas chromatography. The absence of parts that deal with packed columns enable the 3500 to offer more flexibility, efficiency, performance, and higher resolution than packed/capillary GCs.

In creating the 3500, Varian have stressed ease-of-use. By eliminating the packed column capability, which does away with several parts, the column connection is made much easier. In doing so, the cross-talk between the column oven and the injector and detector ovens has also been reached. Self-testing features of Varian's Models 3300 and 3400 GCs have been incorporated in the 3500.

New features of the 3500 include electronic pressure read-out, which allows for easy reproduction of flows specifically for split/splitless injection. The 3500 is the only GC offering split flow read-out and column (calculated) flow read-out.

The 3500 automatic column standby temperature, which eliminates daily flushing and saves start-up time, and heated pneumatics. A rapid detector electronics time constant of 50 ms is seven times faster than other systems and a new ECD, allows the use of hydrogen as carrier gas.

In addition to the new capabilities,the 3500 borrows a number of features from the Varian Model 3300 and 3400 GCs, including its compact size, automatic storage of four complete analytical methods, built-in power failure protection of memory base, self-diagnosis which reduces service requirements, a built-in printer/plotter, rapid column oven heat-up and cool-down, automatic multilinear temperature programming for faster analysis, and easy access pneumatics.

The 3500 is compatible with Varian's new 600 Series Data Systems for total system automation. Another new Varian system, the 8035 Auto-Sampler, can accommodate all injection modes, including on-column capillary injection. The 8035 has advanced capillary features, including control of injection speed, and control of needle residence time for the most accurate results.

An optional built-in, space-saving printer/plotter enables the user to automatically document and confirm the validity of each analysis. The instrument plots the chromatogram, prints the entire analytical method used, and is able to produce a complet RUN LOG of the analysis.

*Details from Varian, 24–28 Manor Road, Walton-on-Thames, Surrey, UK. Tel.: 09 322 43741.*

**Circle No. 24 on Reader Enquiry Card**

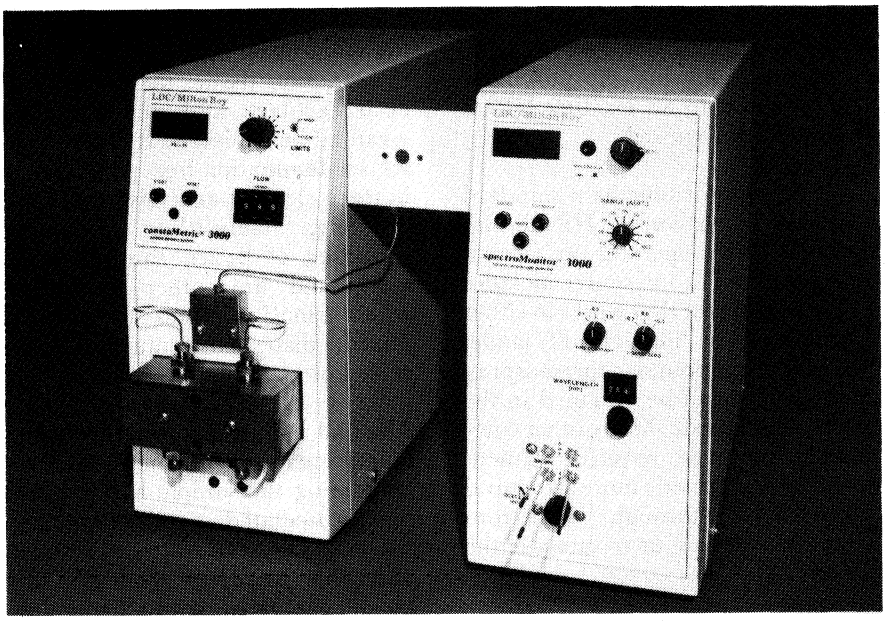

*LDC/Milton Roy's Series 3000 – a modular, high performance liquid chromatography system. The 3000 is built around two new instruments – the constaMetric 3000 solvent delivery system, and the spectroMonitor 3000 variable wavelength detector. Included in the system are a Rheodyne 7125 injection valve, 15 cm C-18 column, and plumbing kit. The constaMetric 3000 provides precise pulseless flow from 0.10 to 9.99 ml/min to pressures up to 6000 psi. The pump can be voltage controlled externally and is pressure monitored. The spectroMonitor 3000 has a wavelength range from 190 to 700 nm, and is designed to handle all aspects of liquid chromatography. A full complement of cell options are available. The vertically designed modules allow for easy system configuration and greatly reduce the amount of bench space required. Further information from LDC, Milton Roy House, High Street Stone, Staffordshire ST15 8AR, UK. Tel.: 0785 813542.*

**Circle No. 25 on Reader Enquiry Card**

### Software for spectroscopy

QUANT-3, Perkin-Elmer's software package for the quantitative analysis of single or multicomponent systems, is now available for ultra-violet/visible and infra-red spectroscopy. Up to 15 components in a mixture can be determined and the results transferred to a printer for a hard-copy report.

QUANT-3 runs on the Perkin-Elmer 7000 Series Professional Computers. The program employs a least squares curve fitting method which can use information from the entire spectrum rather than from a few selected frequencies. This approach has several advantages over traditional methods based on the heights or areas of individual absorption bands. It gives better precision because more information is used in calculating the concentrations. The development of analytical methods is much easier because it is not necessary to select specific frequencies for peak and base-line measurements. Methods are stored on disk so that analyses can be initiated with a single command.

Results can be presented in a variety of formats, including deviations from nominal concentrations and comparisns with a range of acceptable values for quality-control applications. For complete flexibility, QUANT methods can be combined with the instrument control and data manipulation facilities of CDS-3 infra-red or CUV-3 UV/VIS spectroscopy software to give fully automated analyses.

QUANT-3 is easy to use. No typed commands are necessary: users simply follow the instructive software for entering standard spectra and calculating concentration values of unknown samples.

*For further information contact Perkin-Elmer Ltd, Post Office Lane, Beaconsfield, Buckinghamshire HP9 1QA. UK. Tel.: 04946 6161.*

**Circle No. 26 on Reader Enquiry Card**

